# Method Based on the *β*-Lactamase PenPC Fluorescent Labeled for *β*-Lactam Antibiotic Quantification in Human Plasma

**DOI:** 10.1155/2016/4307987

**Published:** 2016-01-24

**Authors:** Max Andresen, Kwok-Yin Wong, Yun-Chung Leung, Wai-Ting Wong, Pak-Ho Chan, Max Andresen-Vasquez, Leyla Alegria, Camila Silva, Pablo Tapia, Patricio Downey, Dagoberto Soto

**Affiliations:** ^1^Departamento de Medicina Intensiva, Facultad de Medicina y Hospital Clínico, Pontificia Universidad Católica de Chile, Santiago, Chile; ^2^State Key Laboratory of Chirosciences, Department of Applied Biology and Chemical Technology, The Hong Kong Polytechnic University, Hung Hom, Kowloon, Hong Kong

## Abstract

Recently, Wong et al. have successfully developed a fluorescent biosensor based on the PenPC *β*-lactamase which changes its intrinsic fluorescence in presence of *β*-lactam antibiotics (BLAs). Here, we studied systematically this correlation among the fluorescence change of the biosensor and the concentration of different BLAs aimed at developing a novel method for estimating the concentration of a wide range of BLAs. This method showed high precision and specificity and very low interference from clinically relevant samples. We were able to monitor the pharmacokinetics of meropenem in healthy volunteers as well as in an ill animal model too, indicating that the implemented method could be suitable for clinical practice.

## 1. Introduction

*β*-Lactams are a class of* antibiotics* which contain a four-membered *β*-lactam ring in their molecular structures. Examples include* penicillins*,* cephalosporins*,* monobactams*, and* carbapenems*. *β*-Lactam antibiotics (BLAs) exert their antibacterial activity by inhibiting the cell-wall-synthesizing function of penicillin-binding proteins in bacteria, thus leading to bacterial death due to osmotic instability [1]. The BLAs are the first line of prevention and treatment in clinical practice against severe infections. Their therapeutic effectiveness and controlled toxicity depend on their adequate dose. In critical care, severely ill patients have profoundly altered their physiology and consequently the pharmacokinetics and pharmacodynamics of BLA among other drugs, limiting the use of parameters previously known for a right dosing thereof. The therapeutic drug monitoring (TDM) has to be used to optimize the clinical effectiveness of BLA in patients with sepsis and septic shock [2, 3]. However, TDM of BLA from human samples is very challenging as a routine practice, because the commonly used instrumental method HPLC is very slow and complex in clinical practice. Therefore, it is highly desirable to develop a simple, reliable, fast, and sensitive method for quantification of BLA in clinical practice.

Wong et al. successfully developed a fluorescent biosensor to detect qualitatively BLA. The fluorescence curves showed an intensity variable as function of the time and also showed a plurality of fluorescent behavior in function of the identity of BLA tested [4, 5]. Here we studied the correlation among the concentration of different BLA and fluorescence behavior of the PenPC biosensor to determine the specificity of the PenPC and the matrix effect from plasma samples over the biosensor. The aim of our work was to develop a method of analysis to estimate the concentration of BLA in plasma using the fluorescence response of PenPC biosensor to BLA.

## 2. Materials and Methods

### 2.1. Chemicals and Reagents

Meropenem was bought from AstraZeneca (Macclesfield, UK), cefazolin was bought from Biosano (Santiago, Chile), and vancomycin (as negative control of BLAs) was bought from Fada Pharma (Buenos Aires, Argentina). All other chemicals were purchased from Sigma-Aldrich (St. Louis, MO, USA): Phosphate saline buffer (PBS): 8 g/L NaCl, 0.2 g/L KCl, 2.72 g/L Na_2_HPO_4_, and 0.24 g/L NaH_2_PO_4_, pH 7.0; dilution buffer: 1% (w/v) BSA in PBS buffer (pH 7.0).

### 2.2. Fluorescent Biosensor Preparation

The E166C mutant of the PenPC *β*-lactamase [(His)_6_-tagged] was produced by the bacterial strain* E. coli* strain BL21(DE3) and purified by Ni^2+^-affinity chromatography according to the previously reported method [6]. The fluorophore labeling of the E166C mutant of the class A PenP *β*-lactamase was performed [5]. Briefly, the E166C mutant was dissolved in 6 M guanidinium hydrochloride solution at room temperature for 30 min to unfold its structure. A tenfold molar excess of fluorescein-5-maleimide was added to the protein solution, and the pH of the mixture was adjusted to 7.5 with 0.2 M NaOH solution. The mixture was stirred at room temperature for 2 h in the dark and then dialyzed against 1 L of 50 mM potassium phosphate buffer (pH 7.0) at 4°C to remove excess fluorescein.

### 2.3. Time-Course Fluorescence Measurements

#### 2.3.1. General

Each trace of time-course fluorescence measurements of standard curve or of the sample assay was carried out in triplicate and then represented as the mean ± standard error.

#### 2.3.2. Standard Curve

For each BLA, a standard curve of at least 7 points among 10^−8^ to 10^−4^ M was built. 10 *μ*L of antibiotic solution of different concentrations (prepared in dilution buffer) was first placed in a black-walled 96-well microplate. Next, 190 *μ*L/well of assay solution (5 × 10^−8^ M biosensor, with 1% (w/v) BSA in PBS buffer (pH 7.0)) was added to each well and mixed thoroughly. Then the fluorescence was recorded along the time.

#### 2.3.3. Assay

Human samples were conveniently diluted. A preanalysis of samples diluted in different ratios was carried out (from 1 : 1 to 1 : 1000) in order to choose the dilution where the fluorescence falls within dynamic range of the technique. 10 *μ*L of diluted samples was mixed thoroughly with assay solution, and the fluorescence was recorded along the time.

#### 2.3.4. Fluorescence Record

The fluorescence signals of the biosensor (*λ*_em_ = 515 nm) were recorded as a function of time using a Synergy 2*®* (BioTek) spectrofluorimeter. Excitation and emission wavelengths were 485 ± 20 nm and 515 ± 20 nm, respectively. The gain factor was set at 75. The sampling time for each well was 1 min, and the time for the whole fluorescence assay was 1.5 h.

### 2.4. Drug Administration and Sample Collections

#### 2.4.1. Ethics

*(1) Healthy Volunteers*. Following the approval from ethics committee of Pontificia Universidad Católica de Chile Hospital in accordance with the Declaration of Helsinki (1964), blood samples were collected from healthy volunteers (*n* = 5). The meropenem pharmacokinetics was tracked.

*(2) Animals Model of Acute Inflammation*. Animal care and experimental design was achieved in agreement with the Guide for the Care and Use of Laboratory Animals, 8th ed. (2011), and with the consent of The Animal Welfare and Ethics Committee of Pontificia Universidad Catolica de Chile.

The meropenem pharmacokinetics was tracked in* Sus scrofa* model of lung injury (*n* = 3) under mechanical ventilation, resembling a critical ill patient with severe sepsis during his treatment in an intensive care unit [7].

#### 2.4.2. Administration of BLA and Samples Collection

For humans a bolus of 500 mg of meropenem was diluted in 100 mL of 0.9% NaCl solution and prepared according to the manufacturer's instructions; it was administered in rapid infusion of 10 min using an infusion pump. For animals the dose was corrected by weight and then meropenem was administered. After the administration, 2 mL of blood samples from human or animals was collected in heparinized Vacutainer tubes at 0, 30, 60, 90, 120, and 180 minutes. The plasma was obtained by centrifuging the blood samples (2.500 rpm, 15 min, and 4°C). The collected plasma samples were frozen at −80°C immediately until they were analyzed.

### 2.5. Statistical Analysis

The nonlineal and lineal fit adjust were made using Prism (GraphPad Software). One-way ANOVA or Student's *t*-test was performed using Prism (GraphPad Software).

## 3. Results

### 3.1. Time-Course Fluorescence Behavior of PenPC with Different BLAs

**Figure 1:**
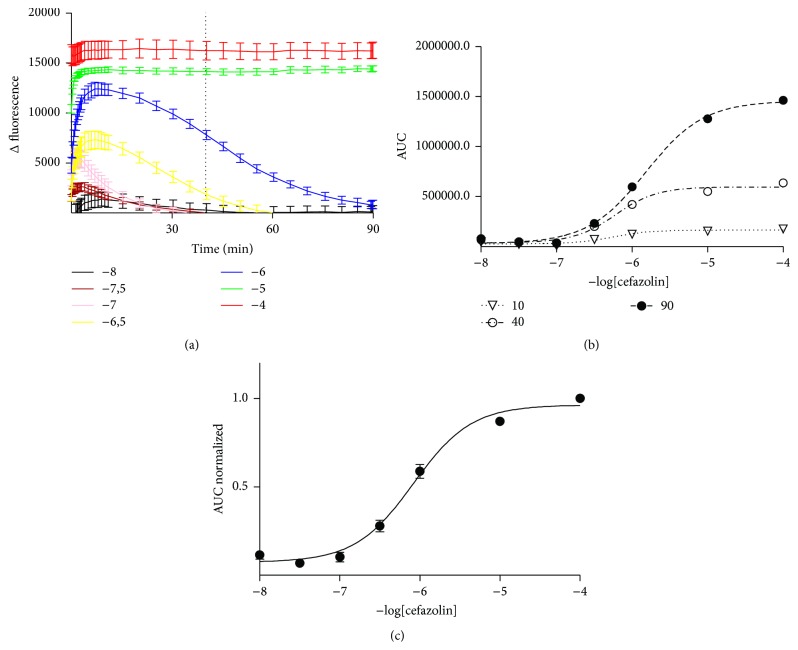
Fluorescence of PenPC induced by different cefazolin concentrations. (a) Time-course fluorescence behavior of PenPC with different cefazolin concentrations. (b) Area under the curve of fluorescence of PenPC induced by different cefazolin concentrations at different time points obtained from (a). (c) Average of at least 3 independent curves of AUC normalized against cefazolin concentration.

**Figure 2:**
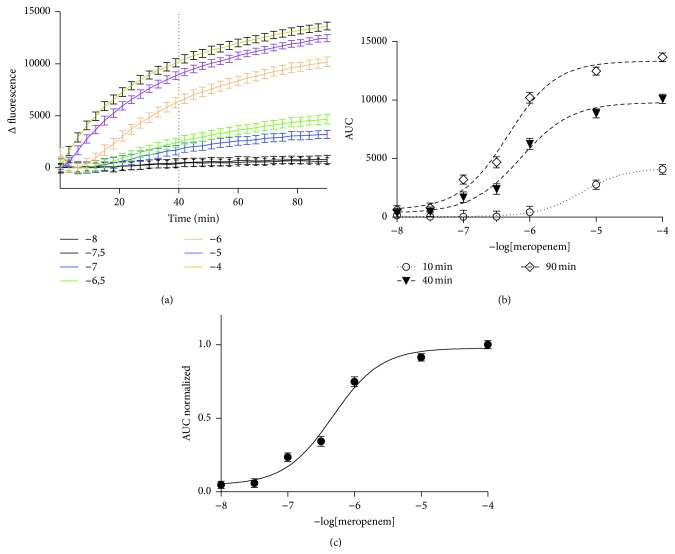
Fluorescence of PenPC induced by different meropenem concentrations. (a) Time-course fluorescence behavior of PenPC with different meropenem concentrations. (b) Area under the curve of fluorescence of PenPC induced by different meropenem concentrations at different time points obtained from (a). (c) Average of at least 3 independent curves of AUC normalized against meropenem concentration.

The fluorescence response of the PenPC biosensor with different concentrations (from 10^−8^ to 10^−4^ M in step of 1 log) of various BLAs was examined. The results show that the biosensor changes its fluorescence as a function of the concentration of BLAs and the fluorescence behavior of such change could be comprised in two specific patterns. The first is a biphasic fluorescence pattern where in low BLA concentrations the fluorescence intensities show a transient bell-like increase, but, at high BLA concentrations, the fluorescence intensity becomes asymptotically stable along the time. Among others, cefazolin followed this pattern (Figure 1(a)). The second fluorescence patterns (response of PenPC to different meropenem concentration) were recorded and fitted to the one-phase association curve. Meropenem followed this pattern (Figure 2(a)).

#### 3.1.1. The Different Pattern of Fluorescence Can Be Analyzed Using the AUC Parameter

Based on the fluorescence response of the PenPC biosensor observed in Figures 1(a) and 2(a), we ruled out use of the parameter changes in fluorescence intensity (CFI) to estimate the BLAs concentration, because it did not follow a unique pattern for the antibiotics tested. In contrast, we observed that the parameter area under the curve (AUC) obtained from the time-course fluorescence followed a unique pattern, independent of the BLAs tested. Thus, we select AUC as the parameter for developing a general method of analysis for a wide spectrum of BLAs.

In a specific time, the AUC along the time showed a pattern that resembles the populations distribution with two possible states (Boltzmann type) suggesting for this case a state represented by the fluorescence emitted by the free biosensor and another state represented by the biosensor associated with its ligand and the consequent change of its intrinsic fluorescence.

The AUC at 10, 40, and 90 minutes derivate from the time-course fluorescence was plotted against the BLAs concentration, cefazolin (Figure 1(b)) or meropenem (Figure 2(b)). Each time could be fitted to a Boltzmann behavior (*r* ^2^ = 0.99).

### 3.2. Assay Optimization

#### 3.2.1. Normalization

In order to allow comparing the values of AUC obtained from identical concentration of meropenem (or cefazolin) either from the same assays (intramethod) or from the independent one (intermethod), each value of AUC was divided by the maximum value of intensity obtained on such experiment by using the saturating BLA concentration. This procedure diminished to less than 5% the variations with respect to the normalized range allowing the comparison between independents experiments (Figures 1(c) and 2(c)).

#### 3.2.2. Matrix Effect of Human Samples

**Figure 3:**
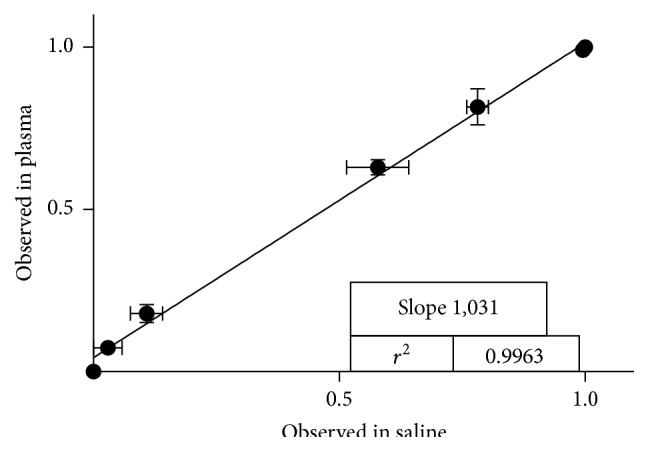
Matrix effect of human samples. The values of AUC obtained from standard curve prepared in saline solution were plotted against the same concentration values of AUC obtained from standard curve prepared in plasma pooled from healthy volunteers (*n* at least 3 independent determinations).

Clinical samples are complex matrices and comprise a plurality of components, such as proteins, lipids, and sugars, which may potentially interact with the sensor. Consequently, the samples could incorporate factors error in the estimation of the antibiotic concentration (e.g., by sequestering the sensor, by components that compete by the substrate, or by introducing other fluorescent components); this unwanted interactions are collectively called matrix effect. In order to evaluate the matrix effects from human plasma on the response of the PenPC biosensor, the fluorescence intensity normalized at different concentrations of meropenem prepared into human plasma was plotted against the value obtained from the same concentration of meropenem prepared in saline solution. The slope value calculated was 1.037, meaning that the AUC fluorescent value induced for any concentration of BLA, prepared in saline solution, was practically equal to that obtained by the same concentration of BLA prepared in human plasma, indicating that the sensor almost did not show matrix effect with plasma components (Figure 3).

#### 3.2.3. Specificity of Assay

**Figure 4:**
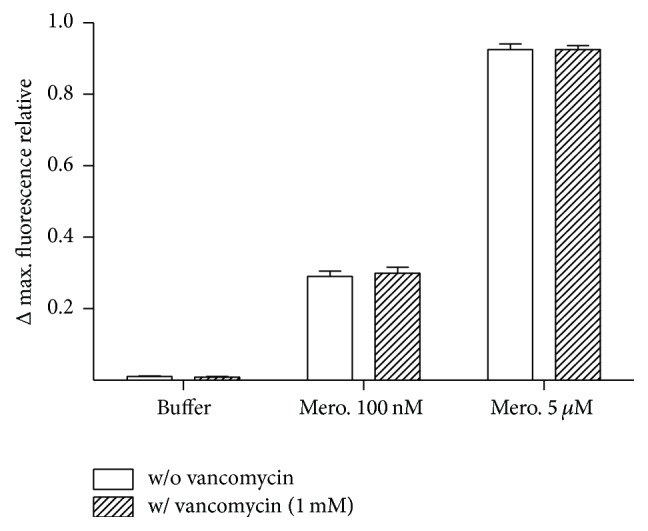
Specificity of assay. The fluorescence intensity of PenPC induced by high (5 *μ*M) and low (100 nM) concentration as well as the absence of meropenem was determined in presence or absence of vancomycin 1 mM. (*n* = 3 independent determinations).

The specificity of the biosensor for *β*-lactam antibiotics was evaluated by a competitive assay using a non-*β*-lactam antibiotic (vancomycin). The fluorescence intensity at the low or high concentration of meropenem was determined in presence or absence of vancomycin. Vancomycin (1 mM) did not induce a significant fluorescence change from the PenPC biosensor. Furthermore, vancomycin did not interfere with the fluorescence response of the biosensor for 100 nM and 1 mM meropenem (Figure 4).

### 3.3. Use of PenPC to Determine Concentration of BLA in Clinical Samples

#### 3.3.1. Plasmatic Pharmacokinetics of Meropenem in Healthy Volunteers Using the Biosensor

**Figure 5:**
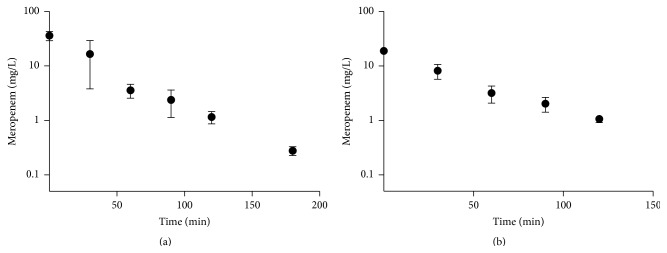
Use of PenPC to determine concentration of BLA in clinical samples. Plasmatic pharmacokinetics of meropenem was determined in (a) human plasma using the biosensor healthy volunteers (*n* = 5) or (b)* Sus scrofa* plasma (*n* = 3) during inflammation process: both of them at the time indicated.

In order to test the feasibility of using this method to determine the concentration of meropenem in human plasma, we carried out a pharmacokinetics test on meropenem in healthy volunteers. The plasma concentration was used to calculate the mean pharmacokinetics parameters of meropenem, using a monocompartmental model. In this case, *C*_max_ of 35 mg/L, a mean half-life of 0.5 h, and a volume of distribution of 0.2 L/kg were obtained using semilog approximation (Figure 5(a)).

#### 3.3.2. Plasmatic Pharmacokinetics of Meropenem in Pig Model of Acute Lung Injury Using the Biosensor

In order to test the feasibility of using this method to determine the concentration of meropenem in plasma under acute inflammation condition, we carried out a pharmacokinetics test on meropenem during a lung injury model in pigs. The pharmacokinetics parameters of meropenem were plotted in semilog presentation (Figure 5(b)).

## 4. Discussion

The fluorescence behavior of the biosensor PenPC induced by different BLAs was correlated with the feature of sensitivity (resistance) to lactamase of each BLA, suggesting a residual enzymatic activity of the sensor. However, here we developed a unique method of analysis, based on the biosensor PenPC, to estimate the concentration of potentially any BLA. Based on the low dispersion of the collected data and the nonsignificant interference by antibiotics without lactam ring, we conclude that the method was reliable and specific. A potential limitation of the method implemented could be its very narrow dynamic range; however, all of the dynamic ranges determined were lower than the minimum inhibitory concentration commonly described for such antibiotics (MIC > 1 mM), so we concluded that the method is suitable for clinical use, and even further the fluorescence of biosensor did not show any detectable matrix effect derived from human healthy model or ill animal model samples. An attractive feature of this method, which is especially focused on performing TDM in critically ill patients, is its high velocity and potential for doing numerous analyses in the same assay. Specifically, it can be carried out in 96-well microplate allowing the parallel determination of 40 samples and 8-point calibration, all in duplicate, in just 40 minutes, opening a full spectrum of possibilities for techniques that require frequent observation of BLAs concentration such as high volume hemofiltration [8, 9]. This study demonstrates the suitability of this biosensor method for future clinical applications, with special application in the intensive care unit, because it provides a simple, reliable, rapid, sensitive, and specific method to detect BLAs in clinical samples.

Although the method developed appears promising for clinical practice, certain limitation still remains to be empirically evaluated. (A) The mechanism of PenPC to change its intrinsic fluorescence is based on its natural ability to interact with beta-lactam ring and apparently prescind from the rest of the molecule. This could lead to overestimating the BLA concentration even when other structural components could be degraded or modified. (B) The components of the samples evaluated did not show important matrix effects. However, the effect of the presence of other beta-lactamases that could interfere with the biosensor still remains to be determined. This ponderation will be especially relevant for determination in samples obtained from septic patients. (C) Finally, the method against a gold-standard technique still remains to be validated. Thus, the next step will be the evaluation of the previous considerations as well as the comparison of the method developed against HPLC.
